# Cognitive rehabilitation in multiple sclerosis: Three digital ingredients to address current and future priorities

**DOI:** 10.3389/fnhum.2023.1130231

**Published:** 2023-02-23

**Authors:** Andrea Tacchino, Jessica Podda, Valeria Bergamaschi, Ludovico Pedullà, Giampaolo Brichetto

**Affiliations:** ^1^Scientific Research Area, Italian Multiple Sclerosis Foundation (FISM), Genoa, Italy; ^2^AISM Rehabilitation Center Liguria, Italian Multiple Sclerosis Society (AISM), Genoa, Italy

**Keywords:** cognitive impairment, cognitive deficit, cognitive disorder, multiple sclerosis, digital twin, telerehabilitation, dual-task, metaverse

## Abstract

Multiple sclerosis (MS) is a neurological chronic disease with autoimmune demyelinating lesions and one of the most common disability causes in young adults. People with MS (PwMS) experience cognitive impairments (CIs) and clinical evidence shows their presence during all MS stages even in the absence of other symptoms. Cognitive rehabilitation (CR) aims at reducing CI and improving PwMS’ awareness of cognitive difficulties faced in their daily living. More defined cognitive profiles, easier treatment access and the need to transfer intervention effects into everyday life activities are aims of utmost relevance for CR in MS. Currently, advanced technologies may pave the way to rethink CR in MS to address the priority of more personalized and effective, accessible and ecological interventions. For this purpose, digital twins, tele-cognitive-rehabilitation and metaverse are the main candidate digital ingredients. Based on scientific evidences, we propose digital twin technology to enhance MS cognitive phenotyping; tele-cognitive-rehabilitation to make feasible the cognitive intervention access to a larger number of PwMS; and metaverse to represent the best choice to train real-world dual- and multi-tasking deficits in virtual daily life environments. Moreover, multi-domain high-frequency big-data collected through tele-cognitive-assessment, tele-cognitive-rehabilitation, and metaverse may be merged to refine artificial intelligence algorithms and obtain increasingly detailed patient’s cognitive profile in order to enhance intervention personalization. Here, we present how these digital ingredients and their integration could be crucial to address the current and future needs of CR facilitating the early detection of subtle CI and the delivery of increasingly effective treatments.

## Introduction

Multiple sclerosis (MS) is an inflammatory neurodegenerative chronic disease with autoimmune demyelinating lesions of the central nervous system (Giovannoni et al., 2016). MS worsens progressively over time and up to 65–70% of people with MS (PwMS) experience cognitive impairments (CIs) [e.g., attention, information processing speed (IPS), learning and memory, and executive functions] (Benedict et al., 2020), with negative impact on personal and social functioning, vocational activities, and quality-of-life (QoL) (Chiaravalloti and DeLuca, 2008). Clinical evidence suggests that CI are present during all MS stages even in the absence of other symptoms (Benedict et al., 2017).

The different clinical phenotypes and the underlying pathological mechanisms play a decisive role in the pattern of cognitive dysfunction (DeLuca et al., 2015). The cognitive profiles of clinically isolated syndrome (CIS) and relapsing-remitting (RRMS) patients are not significantly different, with a prominent slowing of IPS; however, CI frequency tends to be higher in RRMS (20–45%) than CIS (18–35%) (Brochet and Ruet, 2019). As expected, in primary and secondary progressive courses, cognitive deficits are more frequent and severe with a larger number of affected cognitive domains, predominantly memory, executive functions, and IPS (Benedict et al., 2020). Only small studies in radiologically isolated syndrome are available and no conclusions on their prevalence are possible (Brochet and Ruet, 2019). MS evolution is accompanied by a progressive tissue damage (i.e., brain lesions and atrophy) (Calabrese et al., 2009, 2013). Neuroplasticity can balance this tissue damage, by acting in favor of keeping brain functioning effectively (Schoonheim et al., 2015), and contrasting CI onset and worsening (Nasios et al., 2020).

Growing evidence showed that cognitive rehabilitation (CR) enhances both functional and structural neuroplasticity, and this enhancement is specifically linked to the trained domains (Prosperini and Di Filippo, 2019). CR aims at reducing CI and improving PwMS’ awareness of cognitive difficulties faced in their daily living (Mitolo et al., 2015). Furthermore, evidence suggests that the CR positive effects may be more widespread, including fatigue, mood, and QoL (Rosti-Otajärvi et al., 2013; Flachenecker et al., 2017).

Despite well-designed studies are still scarce and efficacy is reported to be low, inconclusive, or preliminary (Rosti-Otajärvi and Hämäläinen, 2014), it is known that CR may be more effective when tailored to a patient’s specific deficit (Sumowski et al., 2018). For this purpose, CI assessment should aim at improving the existing taxonomy of cognitive phenotypes toward more defined and individual cognitive profiles (Sumowski et al., 2018).

Despite the relevance of CR in MS, access to treatment may be precluded for many PwMS due to various and multiple reasons (e.g., inadequate infrastructures, traveling cost, and physical impairment) (Tacchino et al., 2017). Also, interventions should be developed to try to reduce burden for healthcare services, patients, and caregivers without decreasing the treatment quality and effectiveness (Ponzio et al., 2015, 2022). Thus, CR might be planned considering alternative options of care with a major role played by technologies, especially those for remote interventions (Tallner et al., 2016; Brichetto et al., 2019).

In addition, the ultimate goal of MS CR is to enable patients to function as adequately as possible in their environment (Podda et al., 2022). The transfer of CR improvements into daily life would require the training in ecological environments with a large variety of tasks reproducing functional everyday life activities. For this purpose, the adoption of different forms of cognitive-motor dual- and multi-task training, eventually using virtual environments, will be crucial to train PwMS in a context similar to that of everyday life (Goverover and DeLuca, 2018; Abasıyanık and Kahraman, 2022).

Currently, advanced technologies may pave the way to rethink CR in MS to address the priority of more personalized and effective, accessible and ecological interventions. In this scenario, digital twins, tele-cognitive-rehabilitation, and metaverse are the main digital ingredients candidates to enhance cognitive phenotyping, accessibility to treatment, and intervention effect transfer to daily life activities (Sumowski et al., 2018). Here, we present how these digital ingredients and their integration could be decisive to address the current needs of CR, facilitating also the early detection of subtle CI and the delivery of increasingly effective treatments (Rao et al., 2017; Kalb et al., 2018; Sumowski et al., 2018; Montalban et al., 2022).

## Ingredient 1: Digital twin for cognitive phenotyping

Cognitive impairment in MS are still evaluated dichotomously, yielding heterogeneous groups of patients with different profiles of widespread deficits. Several criteria (e.g., impairment in at least two cognitive domains) well define CI in PwMS at group-level, impaired or non-impaired. However, due to a consistent inter-individual variability, less is known about CI prevalence and expression at patient-level (Sumowski et al., 2018). To date, this is the major obstacle for actually understanding the CI causes, identifying individuals at highest cognitive decline risk and obtaining wide consensus on the best approach for MS CI treatment.

Existing taxonomies of predominant cognitive phenotypes led to an increased knowledge of CI in MS. However, enhancing cognitive phenotyping toward individual cognitive profiles may give hope for more effective tailored cognitive interventions for PwMS.

### Cognitive phenotypes in MS

Recently, Leavitt et al. (2018) administered tests of IPS and verbal/visual memory to RRMS patients to evaluate the proportional representation of four cognitive phenotypes: non-impaired, only impaired in IPS, only impaired in memory, impaired in IPS + memory. About 45% of the sample showed at least a CI with prevalence in memory (18.8%) followed by IPS + memory (17.2%) and only IPS (7.8%). Differently, Zurawski et al. (2020), analyzed a sample characterized by the cerebral functional system score of the Expanded Disability Status Scale (EDSS) ≥3 and the other EDSS functional systems scores ≤2. The subjects were also administered tests for intelligence, attention/executive functions, language, memory, visuospatial ability, and IPS. About 80% of the sample had CI in at least one domain (i.e., memory, attention/executive function, and IPS) and 28% showed severe range of cognitive deficits.

Depression and anxiety are the most important confounders of CI because they contribute to early manifestations of cognitive difficulties and, for this reason, should be considered in the early CI diagnosis and cognitive phenotyping (Shahrbanian et al., 2015). Indeed, Podda et al. (2021) identified distinct MS cognitive phenotypes based on both cognitive and mood (i.e., anxiety and depression) domains. The analysis identified four predominant cognitive phenotypes with different patterns of impaired cognitive functions and mood disorders: only memory (28.3%); memory, language, and attention (18.8%); memory, language, attention, IPS, and executive functions (31.7%); and memory, language, anxiety, and depression (21.2%).

Although these studies use different sample selection strategies and analysis methods, the results seem confirming the existence of groups with multi-domain CI and, more interestingly, would suggest the need to consider other relevant dimensions besides mood (e.g., fatigue, pain, or sleep disturbance) for a more complete and accurate taxonomy. However, more descriptive, frequent and rich data could represent the basis to better detect the individual cognitive profile with several implications for clinical practice (e.g., supporting clinical management and decision-making, and planning effective personalized treatments) (De Meo et al., 2021).

### Detecting MS cognitive phenotypes through digital twin technology

Cognitive impairment in MS are usually assessed and monitored through MS-specific paper-and-pencil cognitive batteries (e.g., BICAMS and MAGFIMS) (Maubeuge et al., 2022). Computer-based tests may provide a viable alternative to the conventional neuropsychological assessment (Kourtis et al., 2019). Mobile apps for tele-cognitive-assessment such as Floodlight, Processing Speed Test, and DIGICOG-MS represent a promising new avenue to digitally assess CI and can be incorporated into standard of care for routine cognitive monitoring (Rao et al., 2017; Montalban et al., 2022).^1^ The assessment will occur more frequently, at home in an unsupervised setting, on multiple cognitive domains, and will be integrated with electronic patient-reported outcomes (ePRO) of other dimensions (e.g., mood) significant for MS (Zaratin et al., 2022).

**FIGURE 1:**
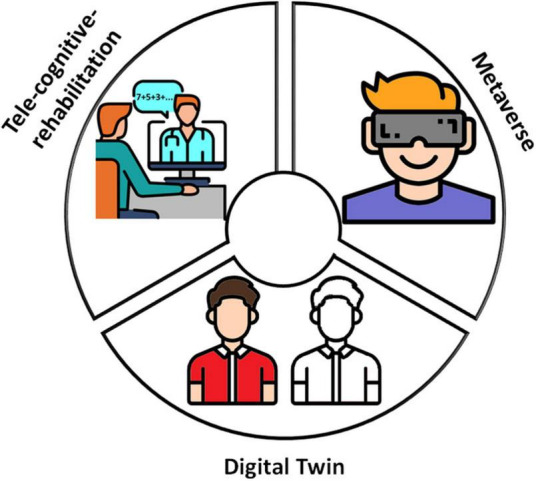
The three digital ingredients. Digital twin, tele-cognitive-rehabilitation, and metaverse are digital ingredients that can improve *per se* cognitive rehabilitation outcomes in MS. In the beginning, data from tele-cognitive-assessment with dedicated apps implementing validated cognitive tests would allow the cognitive phenotyping of PwMS at group-level; over time, based on an increasing amount of data the AI algorithms would provide a rough cognitive virtual patient’s copy. Asynchronous and, especially, synchronous remote interventions can be delivered through tele-cognitive-rehabilitation; the availability of digital tools for remote communication allowed overcoming barriers that limit the CR access to many PwMS and, consequently, starting the treatments as soon as possible and continuing them during the different disease phases. Metaverse makes available virtual scenarios in which PwMS can actually train in ecological environments a plethora of dual- and multi-tasks and everyday life activities. This figure has been designed using modified free images from Flaticon.com.

The availability of long-term longitudinal multi-domain big-data will allow applying the digital twin technology to MS cognitive phenotyping, where the analysis of large amounts of data through new technologies like artificial intelligence (AI) enables visualization of a virtual copy (twin) at different stages of cognitive decline (Figure 1). Cognitive phenotyping based on this revolutionary technology will lead to enhance the detection and treatment of CI at patient-level. Indeed, it will allow detecting early cognitive onset and progression, simulating tailored interventions in advance, predicting potential treatment effectiveness, supporting decision-making and, as consequence, significantly improving QoL and, finally, extending health span (Voigt et al., 2021).

## Ingredient 2: Tele-cognitive-rehabilitation for accessible cognitive interventions

Early cognitive rehabilitative interventions in MS are critical to achieve satisfactory results in maintaining and improving performances (Prosperini and Di Filippo, 2019). Systematic reviews have shown the potential superiority of CR over drugs to improve cognition in MS. The restorative and compensatory approaches, both adopted in MS, have shown moderate-to-large therapeutic effects on PwMS (Benedict et al., 2020).

Cognitive rehabilitation programs should start as soon as possible, be intensive and prolonged, and continue during the different disease phases. However, only recently the availability of digital tools for asynchronous and synchronous remote interventions allowed overcoming barriers that limit the CR access to many PwMS.

### Computer-based CR in MS

In the last decade, several computer-based tools have been developed (Yeroushalmi et al., 2020) and represent an effective method and a great opportunity to prevent unwanted outcomes in PwMS (Charvet et al., 2017). Digital solutions for CR usually implement difficulty levels adaptation and intensive training that reflect in personalized interventions and enhanced cognitive outcomes, sometimes even better than those obtained following traditional approaches (e.g., in attention, episodic, visuospatial and working memory, IPS, and executive function) (Cerasa et al., 2013; Tacchino et al., 2015; Pedullà et al., 2016; Messinis et al., 2017).

In this scenario, telerehabilitation emerged to provide home-based CR interventions eventually adopting novel technologies able to interact with other digital supports, such as wearable biosensors and mobile devices (Xiang and Bernard, 2021). Telerehabilitation represents an alternative efficient method to overcome barriers that prevent PwMS from access to long-term regular rehabilitative interventions (e.g., distance, transportation, disability, and scarcity of services) and to deliver effective treatments in a setting matching with patient’s circumstances (e.g., at-home), priorities (e.g., during lunch break), and abilities (e.g., physical impairment) (Charvet et al., 2017).

To date, most of MS telerehabilitation experiences are asynchronously delivered. Asynchronous systems decouple the components of the interaction so that they occur at different times. As consequence, the patient-therapist connection is delayed by limiting the potentialities of exchanging information, facilitating education, creating common goal setting and treatment planning and, over time, reducing adherence-to-treatment and effectiveness (Wang et al., 2016).

### Enhancing MS cognition with synchronous tele-cognitive-rehabilitation

One main challenge of tele-cognitive-rehabilitation in MS is the definition of a home-based treatment tailoring CI severity and personal needs that could be better defined through approaches based on synchronous supervised interventions, pure or integrated with asynchronous approaches. The constraints imposed by limited bandwidth availability, which prompted the development of asynchronous telerehabilitative services have now largely fallen away, so it is happening a progressive migration to a synchronous approach (Wilson and Maeder, 2015).

The major advantage of a synchronous approach is the immediate patient-therapist interaction that allows refining details and, in many cases, providing clinical decisions or advices within the session, a crucial aspect for more efficient and effective CR (Wilson and Maeder, 2015). After all, the psychologist could benefit of several digitized metrics ranging from conventional exercise scores (e.g., time to perform a task, number of missed targets) to more complicated indexes of lower-level data monitoring user’s interaction (e.g., screen touching) (Serrano-Laguna et al., 2017). Noteworthy, during the same session the psychologist could follow more patients simultaneously, which is time-efficient and cost saving.

Tele-cognitive-rehabilitation should become the standard way of working to quickly overcome barriers to CR in MS (Prvu Bettger and Resnik, 2020; Figure 1). Moreover, numerous recent studies have proven that AI can be used to enhance the quality and efficiency of healthcare services; however, the adaptation of AI in the field of rehabilitation is lacking. The integration of AI-based decision support systems in the tele-cognitive-rehabilitation platforms will allow the psychologist to dispose of routines for the (semi-)automatic personalized exercises selection, which could enhance the cognitive interventions tailoring (Li et al., 2022).

## Ingredient 3: Metaverse for everyday life activities training

Often, PwMS report difficulty to perform multiple tasks simultaneously as required in everyday life activities (e.g., cooking while having a conversation) as consequence of cognitive-motor and cognitive-cognitive interference (Sumowski et al., 2018; Morelli and Morelli, 2021). Difficulties with dual-and multi-tasking have been related to higher risks of falls and worsened QoL (Castelli et al., 2016; Etemadi, 2017).

Cognitive rehabilitation programs should target dual- and multi-tasks performance with specific interventions to address these negative outcomes. The availability of devices for immersive reality may allow firstly training PwMS dual- and multi-tasks conditions, secondly simulating real-world activities in ecological environments, and finally transferring CR improvements into daily life.

### Dual-task training in MS

Recently, Sosnoff et al. (2017) reported a trend for greater gait speed under dual-task conditions after a dual-task training (DTT) compared to a single-task training (STT). Veldkamp et al. (2019) trained PwMS by using CMI-APP (Tacchino et al., 2020), a tablet-based app to deliver cognitive tasks while walking or stepping on the spot; although significant improvements after both DTT and STT, improved dual-task performances were only found in the DTT group. Jonsdottir et al. (2018) showed that a 4-week DTT on treadmill while performing cognitive tasks was significantly effective in augmenting gait resistance and mobility with respect to only supervised muscle resistance training. Overall, these studies also suggested that PwMS may benefit from DTT to improve cognitive functions. However, it seems limited to executive functions, as shown by Van Geel et al. (2020) in PwMS receiving a 10-week choreo-based dance intervention. Indeed, to date, non-significant improvements in IPS and working memory have been found after a DTT. These findings should be interpreted with caution due to the low number of studies with small sample size and variation in the motor-cognitive components.

To our knowledge, no evidences are available on the effect of DTT in immersive environments with augmented feedback (e.g., metaverse), that would allow patient to better perceive the intervention goals, develop a positive perception of their own performance and contextually improve wellbeing and the compliance with long-term care (Maggio et al., 2019). These digital devices will allow disposing of environments customizable on patient-reported needs in order to assess and train patients in multiple and different situations and, as much as possible, to be near to their real-world context.

### Training dual- and multi-tasks and everyday life activities with metaverse

Metaverse application in medicine is still in its infancy. Nevertheless, the patient’s immersion in the metaverse has a wide range of application scenarios and could be useful for CR (Zhou et al., 2022). Its core is the extended reality (i.e., virtual, augmented, and mixed reality), and its main feature is the fusion between virtual and physical worlds (Zhou et al., 2022).

Although a limitation is that the ideal candidates to train with metaverse must have spared cognitive function (i.e., mild or moderate CI) as well as the ability to interact with technologies, the metaverse could intervene in CR already at the early stages of cognitive decline to train brain functions, stimulating neuroplasticity, repairing memory damage, and delaying deterioration (Zhou et al., 2022).

In this context, the metaverse represent the best choice to train cognition in real-world dual- and multi-tasks and to assess cognitive domains trained with specific DTT (Abasıyanık and Kahraman, 2022). The development of a large number of “deep feeling of presence” environments would allow dual- and multi-tasking training in all-around immersive situations (e.g., texting while shopping) without leaving home, tailored on the patient-reported real-world cognitive deficits (e.g., cooking while having a conversation) and eventually enriched with the opportunity to interact with other connected persons (e.g., relatives, friends, and rehabilitator) and elicit emotions (e.g., happiness, surprise, and disgust). Thus, metaverse will allow overcoming the constraints of real environments (e.g., laboratory, outpatient clinic, and at-home) to dual- and multi-tasking assessment and training (Figure 1) and could represent a “backdoor” to improve performances in real-world life.

## Rethinking CR mixing the three digital ingredients

**FIGURE 2:**
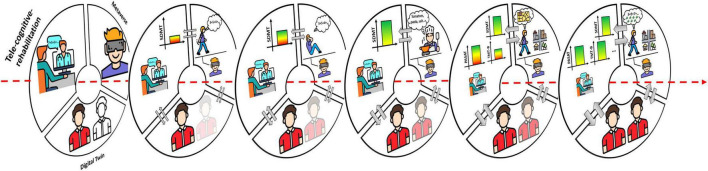
Integration and development of the three digital ingredients. The three digital ingredients represent different sources of data that can be integrated. Over time, the increasing exchange of the data between the different data sources (gray arrows) will feed the AI algorithms that, in turn, will enhance the cognitive phenotyping and provide increasingly defined digital twins. At the same time, the rehabilitative protocols delivered through the tele-cognitive-rehabilitation platform will be increasingly tailored, eventually in a (semi-)automatic way, with a corresponding improvement of the treatment effects (e.g., firstly IPS improved and, then, also other cognitive domains). In the metaverse, the complexity of the tasks increases toward training cognitive functions in more ecological environments and improve the performances transfer into everyday life activities. This figure has been designed using modified free images from Flaticon.com.

Digital twin, tele-cognitive-rehabilitation, and metaverse can contribute *per se* to improve cognitive outcomes in PwMS (Figure 1). However, the fusion of their potentialities could pave the way toward rethinking CR in MS with the final goal to deliver more effective and accessible tailored interventions and to promote the transfer of treatment effects into functional everyday life activities (Figure 2).

In the beginning (Figure 1), AI algorithms are applied to a limited amount of data from apps for tele-cognitive-assessment and ePRO by providing cognitive phenotyping at group-level and, at patient-level, a rough cognitive virtual patient’s copy. Tele-cognitive-rehabilitation platforms will allow the psychologist to set the intervention in terms of exercises, sessions number, session duration, and methods of response (e.g., mouse vs. touchscreen); the protocols will mainly rely on the psychologist’s experience and will be continuously refined across the sessions. Metaverse will provide interactive virtual environments implementing cognitive tasks of different complexity (e.g., counting backwards and making sum) performed during the execution of motor tasks (e.g., walking on a treadmill, steps-on-the-spot, and reaching-to-grasp).

Data from tele-cognitive-rehabilitation (e.g., metrics such as reaction time, accuracy, exercises scores, number of errors, number of executed exercises, etc.) and metaverse (e.g., metrics such as scores, type of errors, physiological and behavioral indexes from sensors, interaction with virtual environments, objects, people, etc.) may be merged with long-term longitudinal multi-domain high-frequency big-data collected through tele-cognitive-assessment.

Over time (Figure 2), the increasing exchange of these data will feed the AI algorithms that, in turn, will enhance the cognitive phenotyping and provide increasingly defined digital twins. The patient’s digital twin will allow identifying and visualizing more accurately how the patient’s cognitive profile are evolving, simulating different treatment options on the virtual patient’s copy in order to test potential real effects, and supporting clinical decisions. The digital twin will also allow providing rules for (semi-)automatic routines to be embedded into the tele-cognitive-rehabilitation platforms for treatment tailoring. In addition, the digital twin will contribute to construct in the metaverse digital scenarios and tasks at increasing complexity, adapted to patient’s abilities, and personalized for the patient’s needs for everyday life activities. The bi-directional link between tele-cognitive-rehabilitation and metaverse will allow on one side supporting the rehabilitative exercises selection to appropriately train on specific cognitive abilities required by the tasks performed in the metaverse; on the other, metaverse would benefit of metrics from tele-cognitive-rehabilitation to establish the exercises difficulty and move toward training complex cognition (e.g., problem-solving, decision-making, thinking, and reasoning).

Mixing the three digital ingredients in terms of data, algorithms, computational models, and platforms leads to dispose of a digital framework for MS CR able to enhance the multi-domain cognitive effects in PwMS and the transfer into functional everyday life activities.

## Conclusion

Current work in MS aims to incorporate advanced technologies into clinics for assessment and treatment of cognitive functions. Deeper knowledge on cognitive phenotypes at patient-level, more accessible and tailored interventions, and better understanding of multitasking deficits in everyday life activities should be considered the main goals by current and future research in MS CR. Big-data, AI algorithms, high computational performances, large bandwidth availability, wearable technologies, and virtual environments are just some of technological advancements that could be necessary to address these goals. Healthcare research is in the midst of a paradigm shift and digital twin technology, tele-cognitive-rehabilitation and metaverse could be the digital ingredients crucial to dispose of more tailored and effective rehabilitative interventions.

## Author contributions

AT contributed to conception of the work, drafting the work, approval for publication of the content, and agreement to be accountable for all aspects of the work. JP, VB, LP, and GB contributed to conception of the work, critical revision for important intellectual content, approval for publication of the content, and agreement to be accountable for all aspects of the work. All authors contributed to the article and approved the submitted version.

## Footnotes

1
https://digicog-ms.aism.it/
